# Screening of SDS-degrading bacteria from car wash wastewater and study of the alkylsulfatase enzyme activity

**Published:** 2013-06

**Authors:** Razieh Shahbazi, Roha Kasra-Kermanshahi, Sara Gharavi, Zahra Moosavi-Nejad, Faezeh Borzooee

**Affiliations:** Department of Biology, Faculty of Basic Sciences, Alzahra University, Tehran, Iran

**Keywords:** alkylsulfatase enzyme, biodegrading bacteria, car wash wastewater, screening, SDS

## Abstract

**Background and Objectives:**

Sodium dodecyl sulfate (SDS) is one of the main surfactant components in detergents and cosmetics, used in high amounts as a detergent in products such as shampoos, car wash soap and toothpaste. Therefore, its bioremediation by suitable microorganisms is important. Alkylsulfatase is an enzyme that hydrolyses sulfate -ester bonds to give inorganic sulfate and alcohol. The purpose of this study was to isolate SDS–degrading bacteria from Tehran city car wash wastewater, study bacterial alkylsulfatase enzyme activity and identify the alkylsulfatase enzyme coding gene.

**Materials and Methods:**

Screening of SDS-degrading bacteria was carried out on basal salt medium containing SDS as the sole source of carbon. Amount of SDS degraded was assayed by methylene blue active substance (MBAS).

**Results and Conclusion:**

Identification of the *sds*A gene was carried by PCR and subsequent sequencing of the 16S rDNA gene and biochemical tests identified *Pseudomonas aeruginosa*. This bacterium is able to degrade 84% of SDS after four days incubation. Bacteria isolated from car wash wastewater were shown to carry the *sds*A gene (670bp) and the alkylsulfatase enzyme specific activity expressed from this gene was determined to be 24.3 unit/mg. The results presented in this research indicate that *Pseudomonas aeruginosa* is a suitable candidate for SDS biodegradation.

## INTRODUCTION

In recent years, surfactants have widely been used in industries and daily life for their interfacial functional capabilities (1, 2). The rapid removal of these compounds from the environment to avoid secondary pollution will make its application safer and more widespread (3).

Synthetic surfactants are components of household and industrial detergents (4). Surfactants are organic chemicals that adsorb at interfaces between air and water or at the interface between oil and water in the case where water is mixed with oil (5). After utilization, large quantities of surfactants and their derivatives are discharged into aquatic and or terrestrial environments (6). These compounds can cause problems in sewage aeration and treatment facilities due to their high foaming capabilities, lower oxygenation potentials and, consequently, kill waterborne organisms (7). SDS, with the chemical formula C_12_H_25_OSO_3_Na, in particular, is an essential component of shampoos, car wash soap and a foaming agent for toothpaste (8). It has two units; (1) a hydrocarbon chain (C_12_) (2) and a sulfate group attached to the chain (8, 9). Due to the increased use of SDS, its bioremediation by suitable microorganisms has gained much importance (10).

Systems involving surfactant, oil and water are being studied due to their high oil recovery potential. Soil washing studies showed greater oil removal with SDS than other surfactants employed. Domestic and industrial wastewater are major sources of pollution and both include a significant amount of surfactant produced by households and diverse industries. Wastewater treatment plants can have operational difficulties with the excess foam generated by these substances (11). The biodegradability of alkylsulfate surfactants in wastewaters, wastewater treatment, marine and estuarine environments is well established (12).

The finding that many bacterial isolates from environmental niches not contaminated by detergents also exhibit alkylsulfatase activity (13) suggests that such enzymes may also play a role in natural environments. Thus, it seems likely that bacteria may be able to mobilize organically bound sulfur for growth, and studies have provided evidence that bacterial sulfatase could play a role in sulfur scavenging. In most cases, degradation of alkylsulfate esters was found to be initiated by alkylsulfatase enzymes that catalyze hydrolytic cleavage of the ester bond to liberate inorganic sulfate. The resulting parent alcohol is further degraded and converted to CO_2_ and H_2_O by β-oxidation or incorporated into cellular lipids (14). The structural genes encoding a potential alkylsulfatase (*sds*A) and putative LysR family transcriptional regulator (*sds*B) of the *sds*A alkylsulfatase gene of strain ATCC19151 have been cloned and sequenced. The *sds*A gene was shown to code for alkylsulfatase as its mutants lacked appropriate enzymatic activity (6).

In this study, SDS-degrading bacteria were isolated from car wash wastewater in Tehran. The isolate was identified by biochemical tests and 16S rDNA partial gene sequencing. Data regarding SDS biodegradation capabilities and alkylsulfatase activity of the isolate as well as the identification of its coding gene is reported.

## MATERIALS AND METHODS

### Screening of SDS- degrading bacteria

SDS-degrading microbial cultures were isolated from wastewater of a car wash located in Tehran by inoculated samples (5%) in 500 ml Basal salt medium containing of KH_2_PO_4_ 3.5 g/l, K_2_HPO_4_ 1.5 g/l, NH_4_Cl 0.5 g/l, NaCl 0.5 g/l, Na_2_SO_4_ 0.14 g/l, MgCl_2_.6H_2_O 0.15 g/l and with the addition of 1.5 mM sodium dodecyl sulfate and the final pH of 7.1 (8, 15). The inoculated media were incubated at 30°C with constant shaking, (150 rpm). After 24 h, the liquid culture was transferred to solidified (2% agar), basal salt medium with 1.5 mM SDS in culture plates. Following three subcultures on solid media, four different bacterial colonies were isolated; characterization and identification of the isolate with the highest biodegradation capability was carried out both biochemically (Table 1) and by 16S rDNA sequencing (8).

**Table 1:** Biochemical characteristics of *Pseudomonas aeruginosa* KGS.

Test	*Pseudomonas aeruginosa*
Motility	+
Oxidase	+
Catalase	+
Growth in 42°C	+
Citrate	+
TSI	k/k
Tryptophanase	-
Fermentation of glucose	_
Pigment production	+

### Methylene blue active substance (MBAS) assay

Concentrations of SDS were determined by the methylene blue active substance (MBAS) assay as described previously. One hundred microlitres of sample was added to 100 ml separating funnel containing 9.9 ml deionized H_2_O followed by the addition of 2.5 ml methylene blue solution (0.5%) and 1 ml of chloroform. The funnel was shaken vigorously for 15 seconds and the combination was allowed to separate followed by the removal of the chloroform layer into a second funnel. The extraction was repeated three times using 1 ml chloroform each time. Prior to adding 5.0 ml of wash solution, all chloroform extracts were combined in the second funnel and shaken vigorously for 15 seconds. The chloroform layer was drawn off into a 10 ml volumetric flask. The wash solution was extracted twice with 1 ml chloroform. All extracts were combined and diluted to the 10 ml mark with chloroform. The absorbance was read at 652 nm against blank chloroform in a quartz or glass cuvette (16, 17).

### Taxonomic characterization of isolated bacteria

The isolate was identified by amplification and the subsequent sequencing of 16S rDNA. *Pseudomonas aeruginosa* KGS genomic DNA was extracted from bacterial colonies by phenol-chloroform method. The 16S rDNA gene from the genomic DNA was amplified by PCR with the following forward and reverse primers of 16S rDNA, f (5′- AGAGTTTGATCMTGGCTCAG-3′) and r (5′- TACGGYTACCTTGTTACGAC-3′). PCR was carried out in a thermocycler (TECHNE, UK) using *Taq* DNA polymerase (Fermentas, Canada). The PCR program consisted of initial denaturation 96°C for 4 min, followed by 35 cycles each of 94°C for 1 min, 61°C for 30 s, 72°C for 50 s; 72°C for 4 min; and incubation at 4°C for 10 min. PCR products were purified with DNA extraction kit (Bioneer, South Korea). Both strands of the PCR product were sequenced by dideoxy chain termination method.

The 16S rDNA gene sequence of KGS was compared with those compiled in the NCBI/EZtaxon/ Ribosomal Database Project (RPD)/ EMBL nucleotide sequence databases using the BLAST (blastn) program (http://www.ncbi.nlm.nih.gov/BLAST/), and all sequences were aligned using the Clustal W program (18). A phylogenetic tree and neighbor-joining phylogeny were constructed using the MEGA software package version 4.0 (19) and bootstrapping was used to evaluate the reliability of the phylogenetic reconstructions (1,000 replicates).

Preparation of enzyme extracts. Bacterial cells were harvested at day 2 by centrifugation of a 12 ml culture at 10,000 × g for 20 min at 4°C in a Beckman J20 high-speed centrifuge. Cell pellets were resuspended in 10 mM Tris-HCl (pH 7.5). The cells were ruptured by sonication (Misonix sonicator, Q55) for a total duration of 30 min, consisting of intermittent pulses of 30 s on and 30 s off. The crude fraction was subjected to ultracentrifugation at 105,000 × g for 2 h and the supernatant was removed for enzyme studies. Protein concentration was determined by Coommassie dye-binding assay at 595 nm using the Bio-Rad™ Bradford reagent (17).

### Alkylsulfatase assay

Alkylsulfatase activity in cell extracts was assayed by incubating 50 µl partially purified enzyme from KGS isolate with 450 µl of 50 mM Tris-HCl and 500 µl of 100 mM SDS. The decrease in substrate concentration was measured by MBAS assay as previously described. Under the assay conditions, the disappearance of SDS followed a linear graph for 15 min. Total enzyme activity was assayed from the initial rates of SDS elimination. One unit of enzyme was determined as the amount of enzyme required to convert 1 µmol of SDS per minute under assay conditions (17).

### Identification of *sds*A gene in bacteria isolated from car wash waste water

Genomic DNA was extracted from bacterial colonies by alkaline lysis (17). The PCR mixture contained 5 pM of each primer, 0.2 mM of each deoxynucleotide triphosphate, 1 × reaction buffer, 50 mM of MgCl_2,_ 1 U of *Taq* DNA polymerase (Fermentas, Canada) to obtain a final volume of 25 µl. The *sds*A gene from genomic DNA was amplified by PCR using the following forward and reverse primers of *sds*A gene (sdsAF 5′-GTCTACACCCACTTCCACCCGG-3, sdsAR 5′-GTGGCGGCAGCTTGACCTTGACCTTCTC-3). PCR was performed under the following conditions: initial denaturation at 94°C for 5min, 35 cycles of 94°C for 1 min, 52°C for 1 min, and 72°C for 1 min, and a final extension at 72°C for 10 min. PCR product was analyzed for identification of sdsA (670 bp) in the bacteria from car wash wastewater.

## RESULTS AND DISCUSSION

### Screening and identification of SDS biodegrading bacteria

**Fig. 1:**
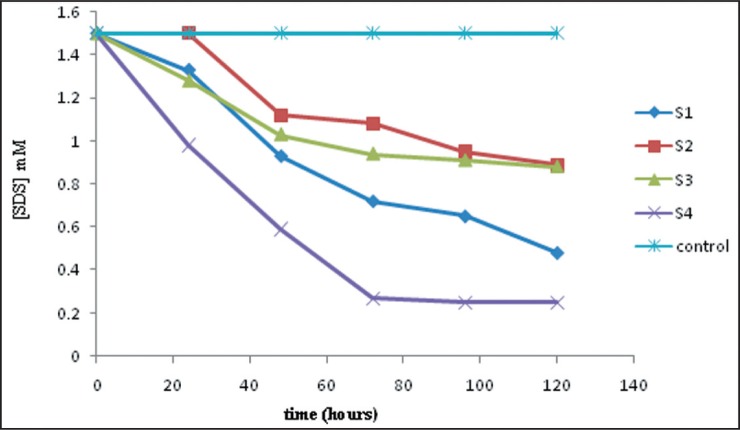
Degradation of SDS by car wash wastewater isolates S1, S2, S3 and S4

Out of the four SDS- degrading bacteria isolated, isolate S4 exhibited a higher capability in degrading SDS with 84% degradation after four days incubations (Fig. 1).

Isolates S1, S2 and S3 exhibited lower potential in SDS degradation with only 68%, 50% and 40% degradation, respectively. A control was also included using an uninoculated medium for which degradation was not observed within the same period of time.

**Fig. 2:**
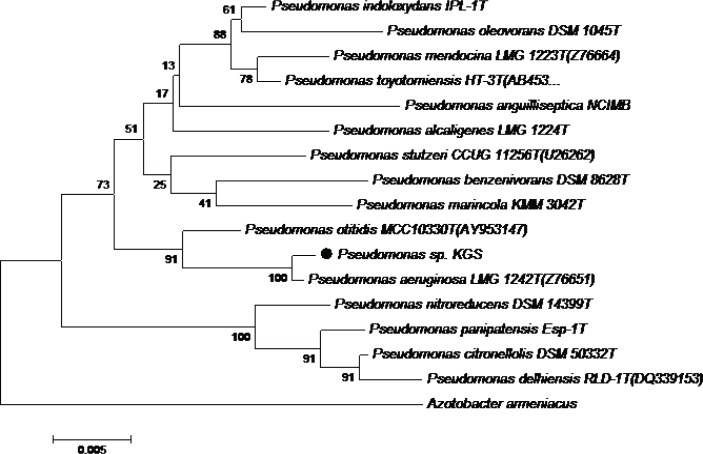
Neighbor-joining tree, based on 16S rDNA gene sequences, shows the phylogenetic relationship between strain KGS and other species of the genus *Pseudomonas*.

The near complete 16S rDNA gene was sequenced (1441 bp) and the analysis clearly demonstrated that strain KGS (S4) is a member of the genus *Pseudomonas* and exhibits maximum similarity with the 16S rDNA sequence of *Pseudomonas aeruginosa* LMG 1242T (Z76651) (100% sequence similarity) (Fig. 2). This sequence has been submitted to the DDBJ/EMBL/GenBank databases under accession No. JQ328193.

Other studies report the isolation of a surfactant-degrading *Klebsiella sp*. from a lagoon contaminated with surfactant (20); other surfactant – degrading bacteria that have been reported are *Nocardia amarae* (21), *Pseudomonas sp*. strain C12B (14, 22), *Comamonas terrigena* (23), *Citrobacter braakii* (24), *Bacillus cereus* (25), *Pseudomonas beteli* and *Acinetobacter johnsoni* (8) and SDS- degrading *Klebsiella oxytoca* is isolated from soil (17).

### Alkylsulfatase activity

Purified or partially purified alkylsulfatases have shown high specific activity about 1-97 U/mg (17, 22, 26) while higher specific activity for purified recombinant alkylsulfatases has been obtained (1.00×10^3^ U/mg) (27). However, various specific activities (about 4 to 90 U/mg) have been reported for alkylsulfatase in crude extracts isolated from microbial sources (28). The variety in the specific activity is related to differences in either the amount of alkylsulfatase activity (amount of enzyme and/or its K_m_) or unit definition (e.g. type of substrate and/or time of activity measurement) (17, 22, 28). In this study, specific activity of alkylsulfatase in crude extract was calculated to be 24.3 U/mg protein, while previous researches have reported specific activity 1.07 U/mg for alkylsulfatase (17). In comparison to the variety of lipase and alkylsulfatase specific activities from different sources, the crude extract has shown relatively high specific activity for alkylsulfatase which makes it suitable for industrial applications (17, 28).

### Identification of alkylsulfatase enzyme (*sds*A) gene

**Fig. 3:**
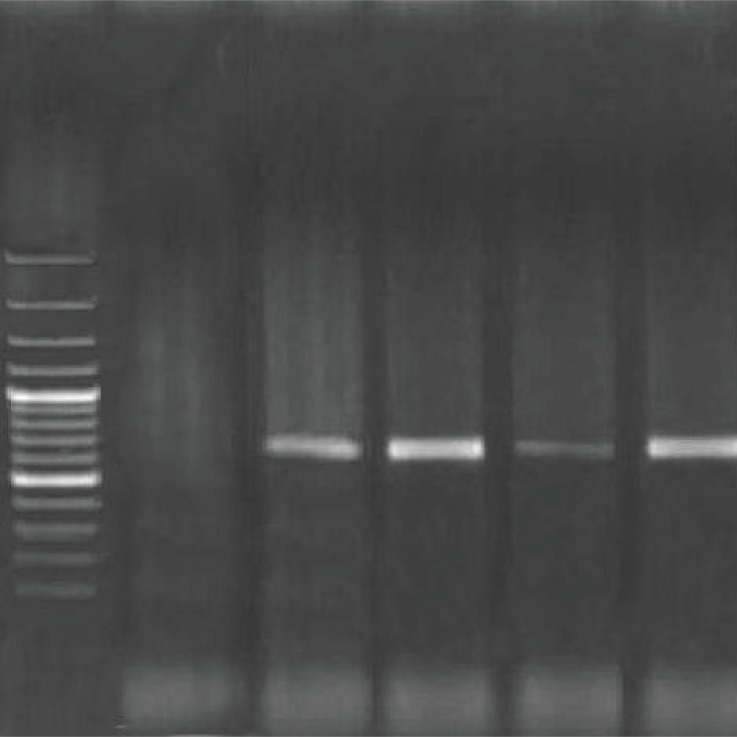
Identification of the *sds*A gene (670 bp) in four isolates from car wash wastewater, respectively from left to right: Molecular weight marker (100bp), negative control, isolate S1, isolate S2, isolate S3, isolate S4 (KGS)

In recent years, molecular techniques have frequently been used to isolate SDS-degrading bacteria by the detection of *sds*A gene (670bp) in all bacteria able to utilize SDS as a carbon source. Our results (Fig. 3) confirm that bacteria isolated from car wash wastewater that were able to degrade SDS, carried the *sds*A gene and consequently, the presence of this gene can be used as a choice indicator for screening of SDS degrading bacteria. The *sds*A gene from strain *Pseudomonas* sp. *ATCC19151* was previously cloned and shown to encode a potential alkylsulfatase (29) having a metallo- β- lactamase domain, which was the first representative of group III of sulfatases (30).

### SDS degradation study

The bacterial isolate from our laboratory was shown to degrade almost 84% of the SDS after four days of incubation. A much higher degradation of SDS (7 mM SDS) has been reported using a consortium *Acinetobacter calcoaceticus* and *Pantoea agglomerans* with complete degradation occurring after approximately 5 days (31); however, the conditions of degradation were markedly different from our study since nutrient broth as a supplement and agitation at high speed (250 rpm) were used (17, 31).

## CONCLUSION

In conclusion, an SDS-degrading bacterium isolated from an SDS-polluted car wash wastewater sample from Tehran has shown valuable biodegrading potentials. The process of characterizing the enzyme involved in SDS degradation is also underway which would further clarify the capabilities of this bacterium in bioremediation processes. The results obtained in our study show maximum degradation of SDS (84%) by *P. aeruginosa KGS* in basal salt medium containing 1.5mM SDS at pH 7.1, temperature 30°C and agitation at 150 rpm after four days incubations. Alkylsulfatase enzyme specific activity of this bacterium was determined to be 24.3 unit/mg and the SDS-degrading isolate was shown to carry the *sds*A gene.
